# A case report of fracture-related infection with *Metamycoplasma hominis* in an immunocompetent patient

**DOI:** 10.5194/jbji-9-271-2024

**Published:** 2024-12-05

**Authors:** Karishma Gokani, Prabu Balasubramanian, Edward Matthews, Dunisha Samarasinghe

**Affiliations:** 1 Microbiology department, Imperial College London Healthcare NHS trust, London, W2 1NY, UK; 2 Orthopaedics department, Imperial College London Healthcare NHS trust, London, W2 1NY, UK

## Abstract

We report a case of post-traumatic *Metamycoplasma hominis* fracture-related infection of the right femur in a young male with no identified immunodeficiency. Treatment required multiple washouts and femoral nail revision, combined with 10 weeks of treatment with doxycycline and clindamycin.

## 1 Introduction

Fracture-related infection (FRI) is a serious complication following internal fixation of fractures. In the context of polytrauma and open injuries, the risk of this complication is significantly increased. There are established diagnostic criteria (Metsemakers et al., 2020). However, there is no consensus on the management of these infections. Often a combination of medical and surgical management with a multi-disciplinary team (MDT) approach is required (Metsemakers et al., 2020; Wong et al., 2022).

*Metamycoplasma hominis* (*M. hominis*) is the smallest known free-living prokaryote. It is difficult to isolate in the laboratory as it is fastidious and has no cell wall, and so it does not Gram stain (McMahon et al., 1990). Colonies are small and translucent and so are easily missed (McMahon et al., 1990). Identification is often by PCR (Chen et al., 2020). *M. hominis* is most commonly isolated from the lower genital tract, and extra-genital infection, including bone and joint infection, is rare and is commonly associated with immunodeficiency (Ahmed et al., 2021).

We report a case of post-traumatic *M. hominis* FRI in a young male with no identified immunodeficiency.

## 2 Case report

A healthy 20-year-old male presented following a motorbike accident. A whole-body computed tomography (CT) scan revealed a Gustilo–Anderson (GA) type-3 open right femur fracture and a right-sided pneumothorax. An intercostal drain was inserted, and the same day he underwent retrograde reamed intramedullary nailing. During the procedure, he became significantly hypoxic, the cause of which was identified as a pulmonary artery embolus on angiography. He was treated with a therapeutic dose of low-molecular-weight heparin.

**Table 1:**
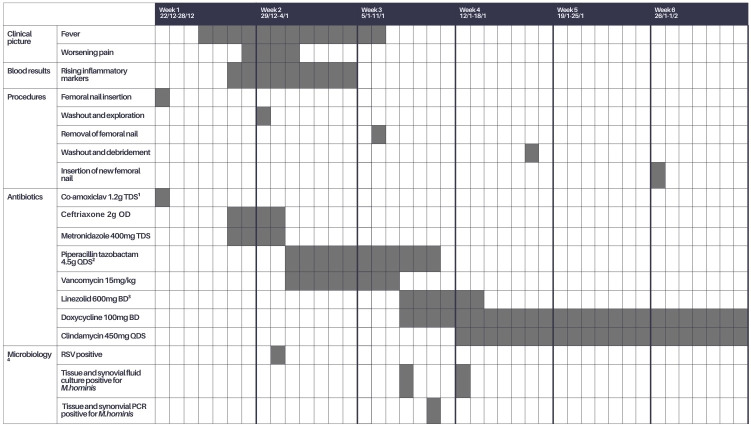
**Table 1**Patient course.

^1^ Prophylaxis as per the trust guidelines of an open fracture. ^2^ Beginning of broadening cover due to persistent fever. ^3^ Vancomycin switched to Linezolid due to unstable vancomycin levels. ^4^ All samples were processed according to local standard operating procedures.

Three days following admission, he developed a persistent fever with normotensive tachycardia and no obvious focus of infection on examination. He had right-thigh swelling consistent with post-operative changes; his wound had no significant erythema, warmth, or exudate; and his pain was stable. Antibiotics were commenced for sepsis of unknown origin according to trust guidelines. A septic screen showed him to be respiratory-syncytial-virus-positive from a throat swab but yielded no other positive results. The following day, his haemoglobin dropped from 105 to 54 g L^−1^, accompanied by worsening thigh pain. CT angiography showed no active arterial bleeding. He received two units of packed red cells, and his anticoagulation was discontinued.

He continued to be persistently febrile with rising inflammatory markers and progressive thigh pain, despite 48 h of broad-spectrum antibiotics. Given these symptoms, he was taken to theatre for tissue sampling and a washout. There was frank haemarthrosis but no pus. Compartment manometry showed normal pressures. Seven tissue samples and one synovial fluid sample were sent for culture and PCR. All the samples were obtained using separate sterile instruments. Synovial fluid microscopy revealed a high number of white cells with 95 % polymorph predominance.

He remained pyrexial and tachycardic a week after the initial washout, so the femoral nail was removed and a temporary external fixator was placed. Large amounts of pus were seen in the proximal locking screw wound. Two pus samples and two tissue samples were sent to microbiology for culture and PCR.

The following day, *M. hominis* was isolated by culture from six tissue samples and the synovial fluid sample from the first washout. Initial identification was from growth on blood agar under anaerobic conditions following incubation in brain–heart infusion. This was confirmed by 16S PCR on both sample types. All four samples from the second washout also subsequently cultured *M. hominis.* His antimicrobial treatment was switched to doxycycline and, following discussion with the *mycoplasma* reference laboratory, clindamycin was added to the regimen.

After MDT discussion, a decision was made to delay definitive fixation of the femur until repeat intraoperative samples were negative and to continue antibiotics for at least 6 weeks. Tissue and fluid samples taken 9 d following commencement of doxycycline were culture-negative after 9 d of incubation, and so a new femoral nail was inserted. The negative samples were taken while the patient was on appropriate antibiotics, so complete eradication of the organism was not certain. However, it was felt that the need for definitive femoral fixation outweighed the risk of residual infection. He was eventually discharged 8 weeks into admission. His inpatient stay is outlined in Table 1.

**Figure 1:**
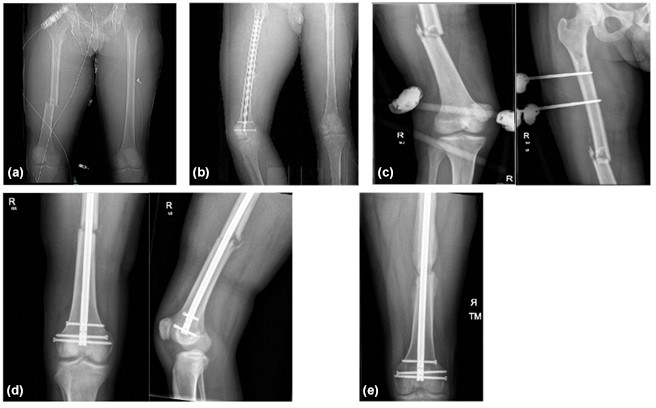
**Figure 1(a)** Initial CT scan showing the femur fracture. **(b)** CT scan following initial fixation with intramedullary nailing. **(c)** Plain radiograph following temporary stabilisation with a knee-spanning external fixator following removal of nail and bony debridement. **(d)** Plain radiograph following revision fixation with removal of an external fixator and intramedullary nailing. **(e)** Plain radiographs at 8-month clinic follow-up showing a bridging callus across the fracture site.

After completion of 6 weeks of targeted antimicrobials, standard radiography showed no signs of healing or callus formation at his fracture, so treatment was extended to 10 weeks, at which point he had signs of healing (Fig. 1). He has continued follow-up for a year, and radiography shows stable non-union with no recurrence of symptoms of infection but ongoing discomfort while mobilising.

Given the rarity of *M. hominis* infection in the immunocompetent, he was referred to an immunologist. HIV and HTLV-1 screening, blood films, lymphocyte subsets, immunoglobulins, and prior infection history were not consistent with immunodeficiency.

## 3 Discussion

FRIs are a significant cause of morbidity and mortality (Wong et al., 2022), affecting up to 30 % of open fractures (Metsemakers et al., 2018). A large prospective cohort study reported a recurrence rate of up to 16 % if treated at 1–10 weeks after injury and resultant amputation in around 3 % of cases (McNally et al., 2022).

Our patient met diagnostic criteria for FRI according to the FRI consensus group given the presence of *M. hominis* in multiple intraoperative samples (Metsemakers et al., 2020).

*M. hominis* is a common commensal of the genitourinary tract and can rarely cause extragenital infections. These are normally associated with immunodeficiency, PJI, and post-natal infection (McMahon et al., 1990; Chen et al., 2020).

*M. hominis* is the commonest *mycoplasma* species isolated in joint infection. In a systematic review of 34 patients with mycoplasmal septic arthritis, 19 had *M. hominis* detected in sterile samples (Chen et al., 2020). All but two cases were associated with immunosuppression or peri-prosthetic joint infection. The remaining two were in patients with an already inflamed joint, one with *Staphylococcus aureus* septic arthritis and the other with gout.

Our case is unique in that our patient was not immunosuppressed, and there was no prior inflammation of the joint. To our knowledge this has not been described before. There have been two similar cases of *mycoplasma* infection following trauma. However, unlike our case, these were cases of septic arthritis associated with bacteraemia (Ti et al., 1982). One recent case report described a pelvic fracture complicated by *M. hominis*. However, this followed a significant urethral injury and was isolated from a subrectus collection (Bethel et al., 2022).

Diagnosis is difficult, both clinically and microbiologically. It is clinically and biochemically indistinguishable from other bacterial causes of bone and joint infection with joint aspirates, showing a raised white cell count with a neutrophil predominance (Chen et al., 2020). *M. hominis* is a fastidious organism, requires specific culture media to grow well, and does not Gram stain. In the case series of mycoplasmal septic arthritis described above, *mycoplasma* or *ureaplasma* species were identified by PCR alone in almost 20 % of the cases (Chen et al., 2020). These factors mean that diagnosis can often be significantly delayed.

*Mycoplasma *are intrinsically resistant to many antibiotics due to the absence of a cell wall. In our case this allowed isolation of the organism despite treatment with broad-spectrum antibiotics prior to sampling. They are usually susceptible to tetracyclines, clindamycin, and fluroquinolones. However, approximately 9 % of the isolates are doxycycline-resistant (Ahmadi, 2020). Unlike other *Mycoplasma* species, *M. hominis* is also usually resistant to macrolides (Ahmadi, 2020; Ali et al., 2021).

The management of FRIs remains challenging due to their varied presentation and high treatment failure rates (Horton et al., 2021). It requires regular specialised MDT input, surgical and medical management, and a prolonged follow-up of at least 12 months (Metsemakers et al., 2020). After discussion with the reference laboratory and reporting of sensitivities, we treated our patient with clindamycin and doxycycline given the low local levels of clindamycin resistance. Treatment length in mycoplasmal joint infections varied in case series from 10 d to 8 months and like FRIs often required a combination of medical and surgical management (Ali et al., 2021). The surgical management of fractures, especially when metal implants are necessary, must be carefully weighed against the risk of infection of that metalwork. While tissue sampling for cultures during antibiotic treatment cannot definitively confirm that an infection has been eliminated, it may be the safest approach when considering the risk of infection against the need to treat the fracture. In line with the literature, our case required five different procedures and 10 weeks of antimicrobials, with follow-up still ongoing and union yet to be established.

There are no data on outcomes following FRI with *M. hominis*. However, one-third of patients had poor functional outcomes following *M. hominis* septic arthritis (Ali et al., 2021), and our case continues to experience discomfort on mobilising a year after his fracture. Whether these poor outcomes are related to post-infectious changes or ongoing low-grade infection is unknown. A systematic review estimated the 1-year all-cause mortality rate for FRI to be 18 % (Wong et al., 2022).

## 4 Conclusion

*M. hominis* is a rare cause of FRI, and although usually associated with immunosuppression, it can be seen in the immunocompetent, in particular following trauma. Mycoplasmal infection should be considered in culture-negative bone and joint infection, and specific culture media and PCR should be employed before exclusion of this as a causative organism. Treatment should be managed with an MDT approach, but we recommend prolonged treatment with clindamycin, quinolones, or tetracycline personalised to the patient, depending on the sensitivity of the isolate or local susceptibility data if available.

## Data Availability

No data sets were used in this article.
